# Excessive fat expenditure in cachexia is associated with dysregulated circadian rhythm: a review

**DOI:** 10.1186/s12986-021-00616-6

**Published:** 2021-10-09

**Authors:** Dufang Ma, Xiao Li, Yongcheng Wang, Lu Cai, Yong Wang

**Affiliations:** Department of Cardiology, Shandong University of Traditional Chinese Medicine Affiliated Hospital, Jinan, 250014 Shandong China

**Keywords:** Cachexia, Circadian clock, Adipose tissue expenditure, White adipose tissue, Brown adipose tissue, Inflammation

## Abstract

Cachexia is a progressive metabolic disorder characterized by the excessive depletion of adipose tissue. This hypermetabolic condition has catastrophic impacts on the survival and quality of life for patients suffering from critical illness. However, efficient therapies to prevent adipose expenditure have not been discovered. It has been established that the circadian clock plays an important role in modulating fat metabolic processes. Recently, an increasing number of studies had provided evidence showing that disrupted circadian rhythm leads to insulin resistance and obesity; however, studies analyzing the relationship between circadian misalignment and adipose tissue expenditure in cachexia are scarce. In the present review, we cover the involvement of the circadian clocks in the regulation of adipogenesis, lipid metabolism and thermogenesis as well as inflammation in white and brown adipose tissue. According to the present review, we conclude that circadian clock disruption is associated with lipid metabolism imbalance and elevated adipose tissue inflammation. Moreover, under cachexia conditions, lipid synthesis and storage processes lost rhythm and decreased, while lipolysis and thermogenesis activities remained high for 24 h. Therefore, disordered circadian clock may be responsible for fat expenditure in cachexia by adversely influencing lipid synthesis/ storage/lipolysis/utilization. Further study needs to be performed to explore the direct interaction between circadian clock and fat expenditure in cachexia, it will likely provide potential efficient drugs for the treatment of fat expenditure in cachexia.

## Introduction

The circadian clock is a key homeostatic regulator that controls many physiological and behavioral responses [1]. The master clock located in the hypothalamus synchronizes multiple peripheral oscillators in most cells of the body to ensure the temporal coordination of behavior and metabolism. At the molecular level, the circadian clock involves transcriptional and translational feedback loops, in which the circadian locomotor output cycles kaput (CLOCK) and brain and muscle ARNT-like 1 protein (BMAL1) complex promotes the transcription of clock genes *Period* (*Per1* and *Per2*) and cryptochrome (*Cry1 and Cry2*). The PER and CRY proteins accumulate in the cytoplasm, and then translocate to the nucleus in the evening to repress their own transcription by interacting with the CLOCK:BMAL1 heterodimer [2]. Additionally, the CLOCK:BMAL1 heterodimer promotes the expression of nuclear receptor reverse ERBα (REV-ERBα) and retinoic acid receptor-related orphan receptor isoforms (RORα and RORγ), which negatively and positively regulate *Bmal1* expression, respectively, by binding to ROR response elements (ROREs) in the *Bmal1* promoter [3–5].

Cachexia syndrome is a complex metabolic disorder that results in depletion of skeletal muscle associated, in some cases, with significant reduced fat mass [6, 7]. The hypermetabolic condition has catastrophic impacts on survival and quality of life for patients suffering from critical illness such as cancer, end-stage heart failure, chronic obstructive pulmonary diseases, and severe burn injury [8]. Currently, although augmenting protein intake, anti-inflammatory drugs are prescribed and select appetite stimulants and exercises are suggested as therapies for cachexia [9], the prevention and management of cachexia are still major challenges to clinicians and patients.

Study performed in gastrointestinal cancer patients have found that cachexia is characterized by preferential loss of adipose tissue and decreased fat cell volume, while muscle mass is less affected [10]. Moreover, adipose tissue expenditure may occur earlier than muscle loss [11, 12]. Cachexia manifested by adipose atrophy is a common and severe complication in critically ill patients admitted to the intensive care unit [13]. Available outcome data of critically ill patients have suggested that obese patients have improved outcomes as compared to the critically patients with healthy body weight, termed “obesity paradox” [14–16]. One explain for this phenomenon is that obese individuals have nutritional “reserves”, which provides substrate during critical illness [17]. The accelerated loss of adipose tissue predicts poorer survival, while maintaining adipose tissue mass may prevent muscle loss and improve patient quality of life and survival outcomes [18, 19].

Increased adipocyte lipolysis and activation of brown/beige adipocytes have been found in adipose tissue of patients with cancer cachexia [20]. Since adipose tissue plays an important role in maintaining energy homeostasis and secreting hormones and adipokines that modulate appetite and nutrient metabolism, alterations in adipose tissue mass can have significant effects on whole-body energy homeostasis [21]. It has been established that adipose tissue activity shows circadian rhythmicity and fat metabolic processes, including adipogenesis, lipid utilization/storage and thermogenesis are under controlled of circadian clock. Disrupted circadian rhythm leads to insulin resistance and metabolic inflammation, increases risk of obesity and type 2 diabetes [22]. These studies have led to increased interest in clock-related treatment strategies, such as “chronotherapeutics”, to mitigate metabolic disorders. Nevertheless, to date, studies analyzing the relationship between circadian misalignment and adipose tissue expenditure in cachexia have been lacking. The causal link between circadian misalignment and adipose wasting in cachexia is interesting to explore, which will provide potential treatment for cachexia.

Given the high number of excellent reviews summarizing impacts of circadian clock in background of obesity, the present review aimed to highlight understanding of the links between circadian clock disruption and adipose expenditure in condition of cachexia. In accordance with this purpose, studies published from 1980 to 2021 were reviewed. Basically, Google Academic, PubMed and Web of Science were used to scan scientific articles. Terms such as “circadian rhythm or circadian clock or biological clock or clock gene” and “lipolysis or lipogenic or energy metabolism or lipid metabolism or energy homeostasis” and “adipose tissue or fat tissue or brown adipose tissue or brown adipocyte” and “inflammation or cytokines” were used as key terms.

## Impaired lipogenesis and storage and elevated lipolysis and lipid utilization contribute to fat expenditure in cachexia

Adipose tissue plays a vital role in whole-body energy homeostasis. In mammals, adipocyte presents in three distinct categories: white, brown and beige. White adipocytes have large unilocular lipid droplets and a small number of mitochondria. They synthesize and store triglycerides in fat droplets and break down triglycerides during periods of energy surplus or demand [23]. Additionally, they synthesize and secrete adipokines and hormones that modulate energy metabolism [24]. Compared with white adipocytes, brown adipocytes have more mitochondria and multilocular lipid droplets. Instead of storing lipids, they convert chemical energy into heat through activating uncoupling protein 1 (UCP-1) expressed in the internal membrane of mitochondria—a phenomenon phenotypically referred to as non-shivering thermogenesis [25]. Through this function, brown adipocytes play important roles in modulating body temperature. Additionally, a third type of inducible “brownlike”, UPC-1-expressing adipocytes have been identified within some white adipose depots. These adipocytes have been named beige. Although they are developmentally related to white adipose tissue, they exhibit functional plasticity in terms of their ability to function like brown adipocytes. Beige adipocytes are rich in mitochondria and express UCP-1. As their name suggests, although they are found in white adipose tissue, they have thermogenic capacity in responds to cold exposure, sympathetic stimuli and hormones to dissipate heat through UCP-1 [26]. In contrast, in the context of a high-fat diet, these beige adipocytes are converted into white adipocytes [27].

Exogenous free fatty acids (FFAs) derived from the diet and those synthesized via de novo lipogenesis in hepatocytes are transported into adipocytes by transport proteins such as cluster of differentiation 36 (CD36) or fatty acid transport protein1 (FATP1). To synthesize triglyceride, three FFAs are esterified to a glycerol backbone, and the triglyceride is processed through the endoplasmic reticulum for storage within the lipid droplet [28]. In response to nutritional conditions, adipocytes increase their size (hypertrophy) to store excessive triglyceride effectively in adipose tissue [29]. However, under conditions of cachexia, lipogenesis and lipid storage capacity are impaired [30]. Adipose tissue from tumor-bearing mice contained shrunken adipocytes that were heterogeneous in size [31]. Expression of key adipogenic transcription factors, such as CCAAT/enhancer binding protein α (*C/EBPα*), CCAAT/enhancer-binding protein beta (*C/EBPβ*), peroxisome proliferator-activated receptor γ (*Pparγ*), sterol regulatory element-binding protein-1c (*SREBP-1c*) and associated target genes involved in lipid synthesis pathways, including fatty acid synthase (*Fas*), acetyl-CoA carboxylase (*Acc*), stearoyl CoA desaturase 1 (*Scd1*), glycerol-3-phosphate acyl transferase (*G3P*) and diacylglycerol O-acyltransferase (*Dgat2*), was significantly decreased [32], leading to reduced lipid accumulation in adipocytes.

**Fig. 1:**
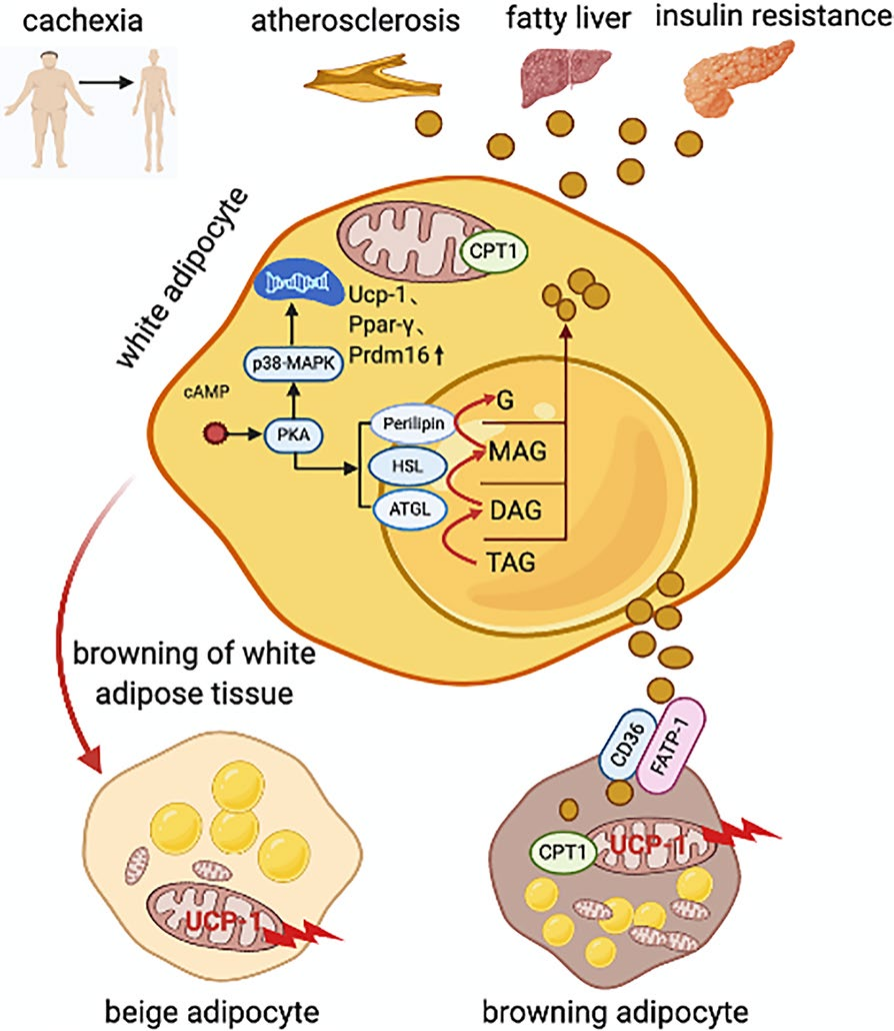
Increased lipolytic activities in white adipocytes and thermogenesis in beige and brown adipocytes contribute to excessive fat expenditure in cachexia. In cachexia, increased catecholamines, corticosteroids and inflammatory cytokines promote lipid lipolysis via active PKA/PKG signaling, causing the hydrolysis of lipid droplets as mediated by ATGL/HSL/Peprelin in white adipocytes. In addition, activated PKA/PKG signaling promotes the transcriptional regulation of white adipose tissue browning as characterized by the upregulated expression of *Ucp-1*, *Pparγ* and *Pdrm16*. This process produces large amounts of FFAs, which contribute to mitochondrial thermogenesis in brown and beige adipocytes, accelerating adipose expenditure. Additionally, excessive FFAs efflux into peripheral tissue or organs leads to ectopic fat deposition, such as that observed in atherosclerosis and hepatic steatosis, causing insulin resistance*. PKA/PKG* protein kinase A/G; *ATGL* lipases adipose triglyceride lipase; *HSL* hormone‐sensitive lipase; *Ucp-1* uncoupling protein 1; *Pparγ* peroxisome proliferator-activated receptor gamma; *Pdrm16* PR domain-containing 16; *FFAs* free fatty acids

Cachexia caused by the hypermetabolic response is characterized by increased lipolytic activities in white adipocytes and thermogenesis in beige and brown adipocytes (Fig. 1). Catecholamines and inflammatory cytokines have been implicated as the primary mediators of this hypermetabolic response in diseases such as cancer and burns [33–36]. They activate their respective receptors on the surface of adipocytes, leading to cellular signaling that activates protein kinase A (PKA) and G (PKG). Then, adipose triglyceride lipase (ATGL), which is a PKA/PKG-activated lipase, initiates triacylglycerol (TAG) catabolism by selectively hydrolyzing the first ester bond, resulting in the formation of diacylglycerol (DAG). Hormone‐sensitive lipase (HSL) preferentially hydrolyzes DAG to monoacylglycerol (MAG), which is further converted to G by monoglyceride lipase (MGL) [37, 38]. This process produces large amounts of FFAs. Excessively increased lipolysis causes FFA efflux into non-adipose tissue, which leads to lipotoxicity in liver, skeleton muscle, pancreas and heart [39, 40]. In cancer patients, increased lipolysis was found to be related to elevated enzyme activities of ATGL and HSL [41]. In tumors-induced cachexia mice models, the increased expression of ATGL contributed to fat depletion and increased rates of lipolysis, while knockout of ATGL protected the tumor-bearing mice from cachexia and loss of fat mass [19]. Similarly, the activated PKA in early cancer induced lipolysis through up-regulation of HSL [42].

Lipolysis-derived FFAs are imported into brown adipocytes and serve as substrate for mitochondrial thermogenesis. In addition, catecholamines and inflammatory cytokines also directly or indirectly, via the activation of PKA/PKG, promote the activity of transcriptional regulators of white adipose tissue browning, including PR domain-containing 16 (PDRM16), PPARγ, and PPARγ coactivator 1α (PGC1α), to induce UCP-1 expression and further induce thermogenesis [43] Recently, inducing “browning” within white adipose tissue has attracted considerable interest in the field of obesity since it shows beneficial metabolic effects by increasing energy expenditure and reducing adiposity. However, it seems to be deleterious in the context of hypermetabolic conditions such as severe burns, end-stage heart diseases and cancer [44–46]; under these conditions, excessive white adipose tissue browning accelerates lipid utilization, ultimately leading to the adipose wasting associated with cachexia [39]. In the colon-26 murine model of cachexia, genes of the fatty acid uptake enzyme lipoprotein lipase (*Lpl*) and the key lipid uptake and transporter protein *CD36* and FFA oxidation *Ppar* were increased. Meanwhile, genes involving thermogenesis such as *p38-MAPK, Pgc1α, Prdm16* and *Ucp-1* were increased. It indicated that increased FFA oxidation and thermogenesis in brown adipose tissue in cancer-induced cachexia [42].

Taken together, as shown in Fig. 1, not only impaired lipogenesis and lipid-storage and increased lipolysis in white adipose tissue contribute to reduced adipose accumulation, but also enhanced thermogenesis induced by activating brown/beige adipose tissue aggravates excessive adipose expenditure in cachexia [47, 48].

## Disrupted circadian rhythm is associated with impaired adipogenesis and lipid synthesis and enhanced lipolysis in white adipose tissue

Circadian clocks in adipose tissue are known to regulate adipogenesis and lipid synthesis. Dysfunctional circadian clocks are associated with abnormal adipogenesis and lipid synthesis. Circadian-disrupted mouse models and adipocyte cell models have been used to independently examine the effects of those aspects (Table 1). For example, *Clock*Δ19/Δ19 mice exhibited increased weight gain related to visceral fat accumulation, and developed hypercholesterolemia and hyperglycemia compared to wild-type littermate controls [49]. In 3T3-L1 preadipocytes, inhibiting *Clock* gene lead to advanced adipogenesis differentiation and lipid synthesis at a very early stage of differentiation [50]. It suggests that CLOCK inhibits adipogenesis and prevents fat accumulation. BMAL1 promotes adipose differentiation and lipogenesis. During the differentiation 3T3-L1 preadipocytes to adipocytes, BMAL1 is highly upregulated. Inhibiting *Bmal1* expression in 3T3-L1 preadipocytes by introduction of siRNA prevented adipogenesis and decreased the expression of lipogenesis genes, such as *PPARγ*, adipocyte protein 2 (*aP2*) and *C/EBP*. Conversely, overexpression of *Bmal1* in 3T3-L1 preadipocytes elevated lipid synthesis activity [51]. Similarly, *Bmal1* overexpression in mouse liver elevated the mRNA levels of lipogenic enzymes and promoted lipogenesis, while *Bmal1* deficiency in hepatocytes displayed decreased levels of de novo lipogenesis and lipogenic enzymes [52]. These findings suggest that BMAL1 regulates adipose differentiation and lipogenesis. The transcription of another clock gene, REV-ERBα has been demonstrated that promotes 3T3-L1 preadipocytes differentiation into mature adipocytes via activation of PPARγ [53]. *Rev-erbα* deficient mice exhibited lipid metabolism disorder, causing increased liver triglyceride and free fatty acid [54]. Treatment of diet-induced obese mice with a REV-ERB agonist reduced triglyceride and free fatty acid in mice [55]. *Cry1* deficient mice (*Cry* − / −) were less likely to gain weight and displayed reduced fat accumulation on a high-lipid diet compared to wild-type littermates [56]. Moreover, knockdown of *Cry1* significantly inhibited the expression of adipogenic markers and lipid droplet formation in 3T3-L1 preadipocytes under adipogenic induction [57]. The *Per3* gene has been found to exhibit robust circadian oscillations in pre-adipocytes and deletion of *Per3* gene significantly increased adipogenesis in vivo [58]. Taken together, it has been convincingly shown that clock genes are involved in adipogenesis by directly or indirectly regulating transcription factors involved in adipogenesis and lipogenesis processes, and disrupted clock genes expression lead to abnormal fat accumulation.

**Table 1:** Summary of chronodisruption-induced metabolic phenotype

Type of chronodisruption	Metabolic phenotype	Reference
**In white adipocyte**		
ClockΔ19/Δ19 mice	Obesity, hypercholesterolemia and hyperglycemia; Circadian rhythm of lipolytic activity were abolished	[49, 61]
Clock-siRNA-infected 3T3-L1 cells	Promote adipogenesis differentiation and lipid synthesis	[50]
Bmal1-siRNA-infected 3T3-L1 cells	Prevent adipogenesis and decrease expression of lipogenesis genes	[51]
Bmal1 − / − mice	Decrease novo lipogenesis; Circadian rhythm of lipolytic activity were abolished; Reduced capacity of fat storage in adipose tissue and increased ectopic fat	[52, 61, 62]
Up-regulated expression of Rev-erbα in 3T3-L1 cells	Promote adipogenesis	[53]
Rev-erbα-deficient mice	Deregulated lipid metabolism	[54]
Cry − / − mice	Reduced fat accumulation	[56]
Cry-shRNA-infected 3T3-L1 cells	Expression of adipogenic markers and lipid droplet formation were inhibited	[57]
Per3 − / − mice	Increased adipogenesis	[58]
**In brown adipocyte**		
Clock mutant mice	Increased lipid accumulation and loss of multilocular appearance in brown adipocytes	[70]
Brown adipocyte Bmal1 KO mice	Disrupted fatty acid utilization in brown adipocyte and obesity	[71]
Bmal1 − / − mice	Increased lipid accumulation in brown adipocytes and dysregulated adaptive thermogenesis; Increased brown adipogenesis and clod tolerance	[72, 73]
Rev-erbα − / − mice	Impaired brown adipogenesis; Disordered rhythms of body temperature and brown adipocyte activity	[74, 75]
Rorα-deficient staggerer mice	Enhanced thermogenesis in brown adipocyte	[76]
Per2-mutant mice	Impaired adaptive thermogenesis	[77]

In addition, circadian clock plays an important role in regulating the rate of lipolysis in white adipose tissue, where lipid is stored in large amounts in the form of triglycerides. To fulfill the day-dependent energy requirement in the human cycles of sleep/wake and fasting/feeding, lipolysis activity is partly mediated by circadian clock in adipoctye. Suzuki et al. [59] firstly observed a diurnal change in the lipolytic activity of isolated epididymal fat cells. Consistently, in humans under constant routine conditions, it has been demonstrated that blood levels of FFAs are directly under circadian control [60]. In obese and/or diabetic individuals, the diurnal rhythm of the lipolysis process is disrupted, as indicated by a study showing that, compared with healthy control individuals, expressions of core clock genes and genes of the canonical metabolic pathways involved in lipolysis lost rhythm in patients with type 2 diabetes [60]. At the molecular level, the key enzymes of lipolysis, including ATGL and HSL, are under the transcriptional control of CLOCK:BMAL1 [61]. In *Clock*∆19 and *Bmal1*^−/−^ circadian mutant mice, the circadian variations of ATGL and HSL were disrupted; in addition, the circadian patterns of serum FFAs and glycerol concentrations and the lipolytic activity in the fat pads were abolished [61]. *Bmal1*^−/−^ mice exhibited reduced capacity of fat storage in adipose tissue, resulting increased levels of circulating FFAs and triglycerides. The increased circulating FFAs level induced the formation of ectopic fat in the liver and skeletal muscle [62]. Therefore, circadian clocks are crucial factors in the regulation of balance between lipid storage and lipolysis.

**Fig. 2:**
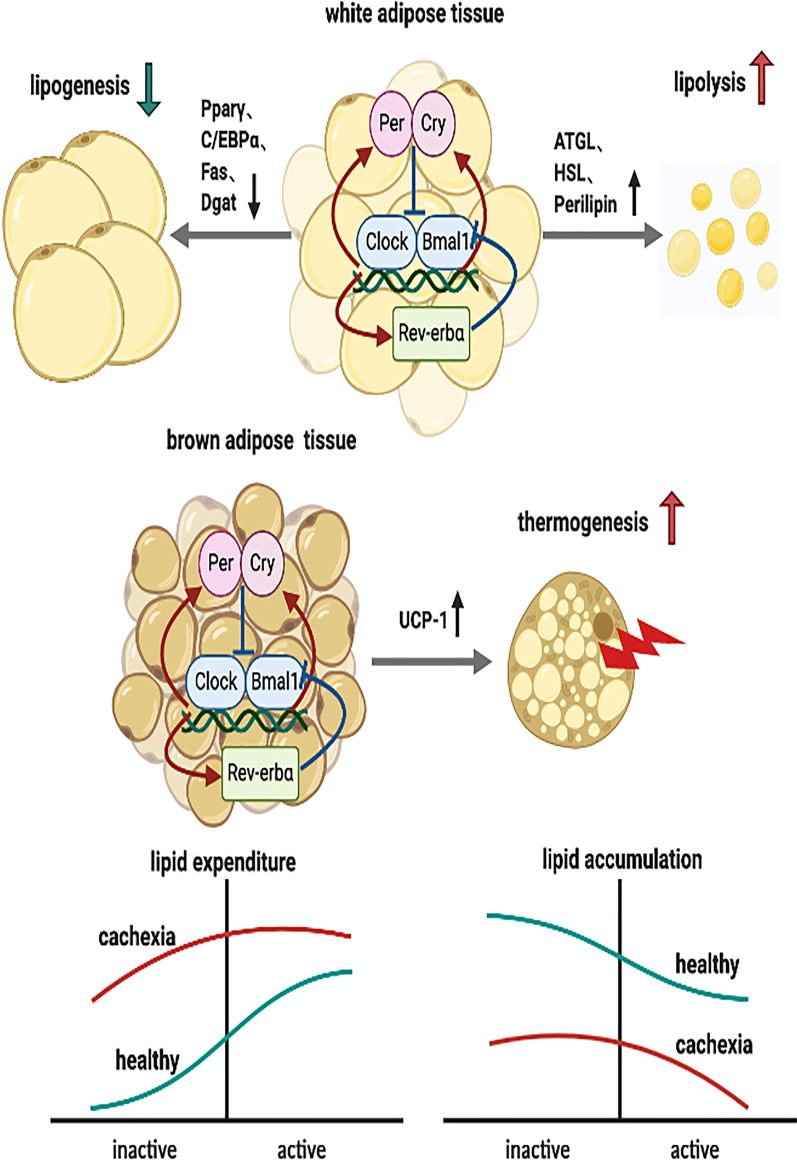
Dysregulated circadian rhythm is involved in lipid accumulation/lipolysis/utilization and thermogenesis in white and brown adipocytes by modulating enzymes involved in fat metabolism. The lipolysis and lipid storage of white adipocytes and thermogenesis of brown adipocytes are mediated by diurnal oscillations controlled by circadian rhythm, while dysregulated circadian rhythm may disrupt the circadian pattern in cachexia. Compared to that in the health condition, in the cachexia condition, the expression of adipogenesis genes, including *Pparγ* and *C/EBPα*, and the lipogenic genes *Fas* and *Dgat* decreased and lost rhythmicity in white adipocytes, indicating decreased lipid accumulation. In addition, the expression of lipases, such as ATGL, HSL and perilipin, and the thermogenesis gene *Ucp-1* increased in white and brown adipocytes during both the day and night cycles, indicating increased lipolysis and lipid expenditure, compared with the expression in the health condition, in both the inactive and active phases. *Pparγ* transcriptional coregulator peroxisome proliferator-activated receptor gamma; *C/EBPα* CCAAT/enhancer-binding protein α; *Fas* fatty acid synthase; *Dgat* diacylglycerol O-acyltransferase; *ATGL* lipases adipose triglyceride lipase; *HSL* hormone‐sensitive lipase

As shown in Fig. 2, in the white adipose tissue of mice bearing C26 tumors, the expression of adipogenesis genes, including *PPARγ* and *C/EBPα*, and lipogenic genes *Fas* and *Dgat* decreased and lost rhythmicity, indicating decreased lipid accumulation in cachexia. Additionally, the protein levels of the lipolytic enzymes perilipin and ATGL were increased at two time points, indicating increased lipolysis during both the day and night cycles. In addition, cachexia resulted in loss of rhythmicity of *Rev-erbα* and *Per2*, and the expressions of *Bmal1* and *Cry1* increased [31]. However, the mechanistic interconnectivity between disrupted circadian clock and reduced lipid synthesis and increased lipolysis in cachexia warrants further investigation.

## Increased fat expenditure in brown adipose tissue is related to disrupted circadian rhythm

Brown adipose tissue is a metabolically active organ distinguished by its unique capacity for adaptive thermogenesis in response to cold and adrenergic stimuli [63]; moreover, its energy-dissipating capacity is necessary to maintain the whole-body energy balance [64, 65]. A study performed by van den Berg et al. [66] revealed that brown adipose tissue displayed a pronounced daily rhythm in FFAs uptake, which was synchronized with the light/dark cycle and was highest upon wakening. Additionally, the diurnal rhythmicity in brown adipose tissue activity determines the rate at which lipids are cleared from circulation, thereby imposing a daily rhythm on plasma lipid concentrations [66]. Indeed, an early study reported that the robust and coordinated expression of circadian oscillator genes, including *Bmal1*, *Per1-3*, *Cry1-2* and *Rev-erbα*, in murine brown adipose tissue [67]; moreover, the oscillations of these clock genes were found to be involved in the modulation of lipid metabolism and thermogenesis processes in brown adipose tissue [68]. Disruption of circadian rhythm by prolonged light exposure decreased sympathetic input into brown adipose tissue and reduced β3-adrenergic intracellular signaling, which concomitantly caused increased adiposity, as indicated by reduced utilization of FFAs and glucose in brown adipose tissue [69]. Thus, impaired diurnal activity of brown adipose tissue contributes to disordered metabolism, while restoring the daily rhythm of brown adipose tissue may be a potential target for the treatment of metabolic disorders.

Like white adipocytes, circadian clock genes have also been demonstrated to be involved in brown adipose tissue activity (Table 1). Whole body *Clock* mutant mice is characterized by excessive lipid accumulation and loss of multilocular appearance in their brown adipocytes [70]. BMAL1 is required for normal lipid metabolism in brown fat. Mice lacking *Bmal1* in brown adipocytes revealed increased lipid accumulation and larger lipid droplets accompanied by dysregulation of genes involved in lipid metabolism and adaptive thermogenesis [71]; moreover, the 24 h rhythmicity of fatty acid utilization was disrupted in brown adipose tissue, and these mice were more prone to diet-induced obesity [72]. Additionally, BMAL1 suppresses brown adipogenesis via direct transcriptional control of key components of the transforming growth factor beta (TGF-β) pathway. Global ablation of *Bmal1* in mice increased brown fat mass and cold tolerance [73]. Conversely, REV-ERBα promotes brown adipogenesis by suppressing key components of the TGF-β, genetic ablation of *Rev-erbα* in mice severely impaired brown fat formation accompanied by loss of brown identity [74]. In regards of brown fat function, REV-ERBα controls circadian thermogenic in a manner that is adaptable to environmental demands through repressing UCP-1 expression in brown adipocyte. Genetic loss of *Rev-erbα* abolished normal rhythms of body temperature and brown fat activity [75]. ROR shares nuclear targets with REV-ERBα, and together with REV-ERBα, it generates the rhythmic expression of BMAL1 that drives the circadian clock cycle [5]. Enhanced UCP-1 and thermogenic gene expression/activity were observed in brown fat of Rorα-deficient mice, indicating repressive effective of RORα on brown fat thermogenesis [76]. PER2 in brown adipose tissue, as a coactivator of PPARα, increases the expression of UCP-1 and promotes the expression of fatty acid-binding protein 3 (Fabp3), a protein important for transporting free fatty acids from plasma to mitochondria to activate UCP-1. These two effects of PER2 were favorable for maintaining the normal body temperature of animals exposed to cold conditions. In contrast, *Per2*-mutant mice are cold sensitive because their adaptive thermogenesis system is less efficient [77]. Thus, the effect of each clock gene on brown adipose tissue is diverse, and the pathophysiological role of circadian clock in brown adipose tissue need to be further explored.

Effects of circadian clock on brown adipose tissue in the background of cachexia is less clear. In brown adipose tissue of C26 tumor-induced cachexia mice, diurnal expression profiling of key regulators of lipid accumulation and fatty acid β-oxidation and their corresponding target genes revealed dramatic molecular changes, indicating diminished de novo synthesis and storage of lipids and increased uptake of lipids for β-oxidation and energy expenditure, as shown in Fig. 2. Increased *Ucp-1*, *Pbe*, and *Cpt1α* expression at specific points coincided with higher brown adipose tissue temperatures during the dark cycle. This result suggested that perturbed diurnal expression patterns of lipid uptake/accumulation/utilization and thermogenesis pathways in brown adipose tissue likely contribute to the fat expenditure in cancer cachexia [78]. However, in the background of cachexia, the direct causal relationship between circadian clock and increased energy expenditure in brown adipose tissue has not yet been systematically explored.

## The circadian clock modulates the expression of chemokines and cytokines involved in fat expenditure in cachexia

Inflammatory cytokines are intimately involved in the initiation and progression of cachexia, with the upregulation of circulating proinflammatory cytokines, including tumor necrosis factor α (TNF-α), interleukin 1 (IL-1), and interleukin 6 (IL-6), as well as decreased anti-inflammatory mediators, including IL-10 [79]. In cancer cachexia, tumor-derived factors such as IL-6 and TNF-α are key drivers of lipolysis and fat depletion [80, 81]. IL-6 is a key inflammatory mediator that upregulates the expression of UCP-1, which mediates white adipose tissue browning during the hypermetabolic response. Mice lacking the IL-6 gene exhibited reduced white adipose tissue browning in response to burn injury [80, 82]. Moreover, IL-6 suppresses lipid synthesis and accelerates lipid catabolism in adipocytes [18]. TNF-α, another critical inflammatory mediator, plays a significant role in cancer cachexia. It promotes lipolysis by increasing the activity of HSL in white adipocytes [83]. More importantly, TNF-α exacerbates macrophage infiltration, which is critical for increasing IL-6 and other proinflammatory cytokines and generating a vicious cycle of macrophage recruitment [84].

The circadian clock controls inflammation and immune functions by modulating the expression of chemokines and cytokines. Circadian clock dysfunction is associated with metabolic inflammation in adipose tissue [85]. CLOCK promotes inflammation by enhancing the genes expression of *IL-6*, *IL-1β*, *TNF-α* and chemokine (C–C motif) ligand 2 (*Ccl2)* [86]. In contrast, BMAL1 is the central mediator regulating the immune system by directly suppressing Ccl2 expression, leading to lower recruitment of inflammatory monocytes into inflamed tissues [87]. Additionally, overexpression of CLOCK leads to enhanced transcriptional activity of the nuclear factor kappa-B (NF-κB) complex, while BMAL1 attenuates the effect of CLOCK on NF-kB, most likely by sequestering CLOCK [88]. This evidence suggests that BMAL1 suppresses, while CLOCK potentiates, the inflammatory response. REV-ERBα prevents the release of IL-6 from macrophages and attenuates Ccl2 by directly binding to the promoter [89, 90]. Time-of-day gating of macrophages isolated from mice with global *Rev-erbα* deletion disappeared, and these mice had increased expression of inflammatory cytokines, including IL-6, Ccl2 and chemokine (C–C motif) ligand 5 (Ccl5), in macrophages [91]. In contrast, a REV-ERBα agonist suppressed the expression of IL-6 and Ccl2 in lipopolysaccharide-stimulated human monocyte-derived macrophages [89]. Two other clock proteins, PER/CRY, constitute another mechanism of clock-mediated inflammation modulation. The global disruption of the clock genes *Per1* and *Per2* exacerbates inflammation [92]. CRY also inhibits inflammation by suppressing the NF-κB pathway, and the absence of CRY results in constitutive elevation of proinflammatory cytokines [93].

Myeloid cells including monocytes and macrophages are the chief cellular effectors of the innate immune system and involved in adipose tissue inflammation during cachexia [94]. In background of burn-induced cachexia, it was shown that polarized M2 macrophages are recruited to subcutaneous adipose tissue and secrete catecholamines to activate browning of white adipose [95]. Ly6C(hi) inflammatory monocytes exhibit diurnal variation, which controls their trafficking to sites of inflammation and polarization. Myeloid cell-specific deletion of *Bmal1* induces expression of monocyte-attracting chemokines and disrupts rhythmic cycling of Ly6C(hi) monocytes, predisposing mice to development of pathologies associated with acute and chronic inflammation [96]. In background of atherosclerosis, *Bmal1* deficiency in monocytes and macrophages promotes atherosclerosis by enhancing monocyte recruitment to atherosclerotic lesions and enhanced M2 to M1 macrophage transformation [97]. REV-ERBα also has a role in regulating inflammation in macrophages. REV-ERBα agonists SR9009 reduced the polarization of bone marrow-derived mouse macrophages to proinflammatory M1 macrophage while increasing the polarization of macrophages to anti-inflammatory M2 macrophages [98]. Moreover, REV-ERBα regulates the inflammatory infiltration of macrophages through the suppression of Ccl2 expression [90].

As discuss below, circadian clock system directly and indirectly regulates the expression and activation state of cytokines and chemokines, and the inflammatory cell migration and transformation. In cachexia, excessive adipose tissue expenditure is closely associated with production of inflammatory cytokines and recruitment of immune cells; however, whether circadian clock dysregulation involves in adipose tissue expenditure through affecting inflammatory cytokines and immune cell is still unknown. In this regard, future studies should determine the association between circadian clock system and inflammation in cachexia-induced adipose tissue wasting.

## Conclusion and future directions

Collectively, a well-functioning circadian clock play important roles in most aspects of adipose biology, from adipogenesis to lipid storage, lipolysis and thermogenesis. Dysregulation of circadian rhythmicity is closely related to disordered lipid metabolism and exacerbated inflammation in adipose tissue, which are critical underlying mechanisms of metabolic disorders.

Nevertheless, our review only provides primary evidences for the link between metabolic disorders and circadian clock dysregulation, studies addressed causes and consequences of circadian clock disruption and adipose expenditure in cachexia are far less understood. In light of the clinical relevance of excessive adipose expenditure and the current lack of effective therapeutic options in cachexia patients, a certain refocusing of clinical and research efforts onto the intricate molecular mechanisms between the dysregulated circadian clock and adipose expenditure in cachexia seems to be necessary and valuable in the future. This field will be to identify circadian clock as a new target in adipose tissues for treating cachexia. Moreover, circadian rhythm-dependent treatment approaches such as “chronopharmacology” and “chrononutrition” should be considered in the treatment and prevention of cachexia.

## Data Availability

Not applicable.

## Abbreviations

ClOCK: Circadian locomotor output cycles kaput
BMAL1: Brain and muscle ARNT-like 1 protein
PER: Period
CRY: Cryptochrome
REV-ERBα: Nuclear receptor reverse ERBα
ROR: Retinoic acid receptor-related orphan receptor
ROREs: ROR response elements
UCP-1: Uncoupling protein 1
FFAs: Free fatty acids
CD36: Fatty acid translocase CD36
FATP1: Fatty acid transport protein 1
C/EBPα: CCAAT/enhancer binding protein α
C/EBPβ: CCAAT/enhancer-binding protein beta
PPARγ: Peroxisome proliferator-activated receptor γ
SREBP-1c: Sterol regulatory element-binding protein-1c
FAS: Fatty acid synthase
ACC: Acetyl-CoA carboxylase
Scd1: Stearoyl CoA desaturase 1
G3P: Glycerol-3-phosphate acyl transferase
DGAT2: Diacylglycerol O-acyltransferase
PKA: Activates protein kinase A
PKC: Activates protein kinase C
ATGL: Adipose triglyceride lipase
TAG: Triacylglycerol
DAG: Diacylglycerol
HSL: Hormone‐sensitive lipase
MAG: Monoacylglycerol
MGL: Monoglyceride lipase
PDRM16: PR domain-containing 16
PGC1α: PPARγ coactivator 1α
FAS: Fatty acid synthase
aP2: Adipocyte protein 2
Fabp3: Fatty acid-binding protein 3
Pbe: Peroxisomal bifunctional enzyme
Cpt1α: Mitochondrial carnitine palmitoyl transferase 1α
TNF-α: Tumor necrosis factor α
IL-1: Interleukin 1
IL-6: Interleukin 6
Ccl2: Chemokine (C–C motif) ligand 2
Ccl5: Chemokine (C–C motif) ligand 5
NF-κB: Nuclear factor kappa-B
